# FLCN and AMPK Confer Resistance to Hyperosmotic Stress via Remodeling of Glycogen Stores

**DOI:** 10.1371/journal.pgen.1005520

**Published:** 2015-10-06

**Authors:** Elite Possik, Andrew Ajisebutu, Sanaz Manteghi, Marie-Claude Gingras, Tarika Vijayaraghavan, Mathieu Flamand, Barry Coull, Kathrin Schmeisser, Thomas Duchaine, Maurice van Steensel, David H. Hall, Arnim Pause

**Affiliations:** 1 Goodman Cancer Research Center, McGill University, Montréal, Québec, Canada; 2 Department of Biochemistry, McGill University, Montréal, Québec, Canada; 3 College of Life Sciences, University of Dundee, Dundee, United Kingdom; 4 Institute of Medical Biology, Singapore, Singapore; 5 Department of Neuroscience, Albert Einstein College of Medicine, New York, New York, United States of America; St. Jude Children's Research Hospital, United States of America

## Abstract

Mechanisms of adaptation to environmental changes in osmolarity are fundamental for cellular and organismal survival. Here we identify a novel osmotic stress resistance pathway in *Caenorhabditis elegans (C*. *elegans)*, which is dependent on the metabolic master regulator 5’-AMP-activated protein kinase (AMPK) and its negative regulator Folliculin (FLCN). FLCN-1 is the nematode ortholog of the tumor suppressor FLCN, responsible for the Birt-Hogg-Dubé (BHD) tumor syndrome. We show that *flcn-1* mutants exhibit increased resistance to hyperosmotic stress via constitutive AMPK-dependent accumulation of glycogen reserves. Upon hyperosmotic stress exposure, glycogen stores are rapidly degraded, leading to a significant accumulation of the organic osmolyte glycerol through transcriptional upregulation of glycerol-3-phosphate dehydrogenase enzymes (*gpdh-1* and *gpdh-2*). Importantly, the hyperosmotic stress resistance in *flcn-1* mutant and wild-type animals is strongly suppressed by loss of AMPK, glycogen synthase, glycogen phosphorylase, or simultaneous loss of *gpdh-1* and *gpdh-2* enzymes. Our studies show for the first time that animals normally exhibit AMPK-dependent glycogen stores, which can be utilized for rapid adaptation to either energy stress or hyperosmotic stress. Importantly, we show that glycogen accumulates in kidneys from mice lacking FLCN and in renal tumors from a BHD patient. Our findings suggest a dual role for glycogen, acting as a reservoir for energy supply and osmolyte production, and both processes might be supporting tumorigenesis.

## Author Summary

The ability of an organism to adapt to sudden changes in environmental osmolarity is critical to ensure growth, propagation, and survival. The synthesis of organic osmolytes is a common adaptive strategy to survive hyperosmotic stress. However, it was not well understood, which biosynthetic pathways and storage strategies were used by organisms to rapidly generate osmolytes upon acute hyperosmotic stress. Here, we demonstrate that glycogen is an essential reservoir that is used upon acute hyperosmotic stress to generate the organic osmolyte glycerol promoting fast and efficient protection. Importantly, we show that this pathway is regulated by FLCN-1, an ortholog of the human tumor suppressor Folliculin responsible for the Birt-Hogg-Dubé cancer syndrome, and by AMPK, the master regulator of energy homeostasis.

## Introduction

Water is a fundamental molecule for life and the ability of an organism to adapt to changes in water content is essential to ensure survival. Hyperosmotic stress promotes water efflux, causing cellular shrinkage, protein and DNA damage, cell cycle arrest and cell death. All living organisms encounter hyperosmotic environments [1,2]. In humans, both renal and non renal tissues are exposed to hyperosmotic stress, a condition that is regarded as a major cause for many chronic and fatal human diseases including diabetes, inflammatory bowel disease, hypernatremia, dry eye syndrome, and cancer [1]. Cells/tissues/organisms have evolved adaptive strategies to cope with threatening hyperosmotic environments [1,2]. Among adaptive strategies, the synthesis of compatible organic osmolytes, which keeps cellular osmotic pressure equal to that of the external environment, is widely used by all organisms [3]. In yeast and *C*. *elegans*, hyperosmotic stress triggers glycerol production via transcriptional upregulation of glycerol-3-phosphate dehydrogenase-1 *(gpdh-1)*, a rate-limiting enzyme in glycerol synthesis [4,5]. Moreover, several osmotic stress resistance mutants of divergent signaling pathways exhibit a constitutive transcriptional upregulation of *gpdh-1*, leading to increased glycerol content [6–10].

Here we define a novel hyperosmotic stress resistance pathway mediated by the 5' AMP-activated protein kinase (AMPK), a key regulator of cellular energy balance [11], which is chronically inactivated by the worm ortholog of the renal tumor suppressor Folliculin (FLCN-1). In humans, *FLCN* is a tumor suppressor gene responsible for the BHD disease, an autosomal dominantly-inherited syndrome associated with increased susceptibility to the development of several cancerous and non cancerous lesions including kidney cancer, pulmonary, renal, pancreatic and hepatic cysts and skin fibrofolliculomas [12–25]. FLCN has been shown to bind AMPK via the scaffold FLCN-interacting proteins FNIP1 and FNIP2 [26,27]. We have recently demonstrated that FLCN negatively regulates AMPK signaling in the nematode *C*. *elegans* and in mammalian cells [28,29]. Moreover, loss of FLCN increased ATP levels via heightened flux of glycolysis, oxidative phosphorylation, and autophagy, which resulted in an AMPK-dependent resistance to several metabolic stresses in *C*. *elegans* and mammalian cells [28,29].

Here we identify a pathway involved in the physiological response to hyperosmotic stress resistance in *C*. *elegans* mediated by FLCN-1 and AMPK. We demonstrate that glycogen is an essential reservoir that is used upon acute hyperosmotic stress to generate glycerol and promote fast and efficient adaptation to prevent water loss and ensure survival. We show that in *flcn-1(ok975)* mutant animals, this phenotype is significantly enhanced, due to the robust AMPK-mediated accumulation of glycogen, which is rapidly converted to the osmolyte glycerol upon salt stress. Our results also suggest that the FLCN/AMPK pathway might be an evolutionarily conserved key regulator of glycogen metabolism and stress resistance.

## Results

### Loss of *flcn-1* confers resistance to hyperosmotic stress in *C*. *elegans*

**Fig 1:**
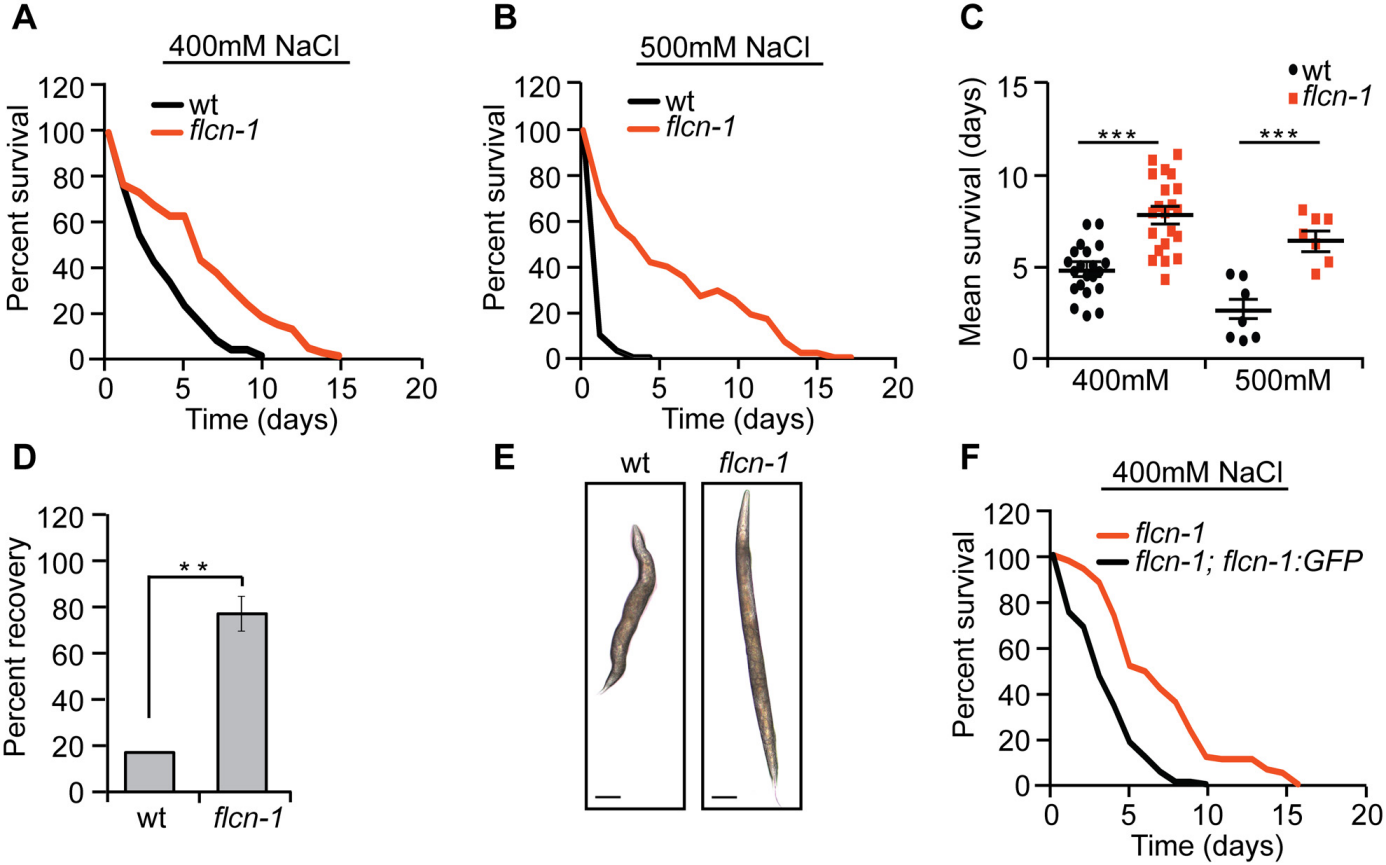
Loss of *flcn-1* confers resistance to hyperosmotic stress. (A-C, F) Percent survival (A, B, F) and mean survival (C) of indicated worm strains exposed to 400mM and 500mM NaCl. (D) Percent recovery from paralysis of wt and *flcn-1(ok975)* animals after 2 hours from exposure to 400mM NaCl. Data represent mean ± SEM, n≥ 3. (E) Representative images of wt and *flcn-1(ok975)* animals treated with 400mM NaCl for 48 hours.

Since we have previously observed that loss of *flcn-1* in *C*. *elegans* increases AMPK-dependent resistance to energy stresses including oxidative stress, heat, and anoxia [28], we asked whether it would also increase resistance to hyperosmotic stress. We measured the survival of wt and *flcn-1(ok975)* animals (S1A Fig) on plates supplemented with 400mM and 500mM NaCl. Loss of *flcn-1* conferred a significant increase in resistance to hyperosmotic stress (Fig 1A and 1B and S1 Table). Although NaCl treatment severely reduced the survival of both wt and *flcn-1(ok975)* animals as compared to untreated animals (Figs 1A, 1B and S1B), the mean survival of *flcn-1(ok975)* animals increased by ~2 and ~3 fold upon treatment with 400mM and 500mM NaCl respectively, as compared to wt animals (Fig 1A–1C). Moreover, we did not observe a significant difference in lifespan between untreated wt and *flcn-1(ok975)* animals, as reported previously [28] (S1B Fig and S1 Table). Importantly, NaCl treatment led to shrinkage and paralysis in both wt and *flcn-1(ok975)* animals. However, *flcn-1(ok975)* mutant nematodes recover significantly faster than wt animals after 2 hours of NaCl treatment suggesting that the mechanism of adaptation to salt is more robust upon loss *flcn-1* (Fig 1D). We also observed a significantly greater number of wt animals with more than 30% reduction of body size as compared to *flcn-1* suggesting that loss of *flcn-1* activates pathways that favor body size recovery after hyperosmotic stress (Fig 1E). Importantly, the hyperosmotic stress resistance phenotype can be rescued by transgenic re-expression of *C*. *elegans flcn-1* (S1 Table and Figs 1F and S1A).

In addition, we used Agilent whole genome *C*. *elegans* microarrays to determine transcriptional profile differences between wt and *flcn-1(ok975)* mutant animals [30]. Key genes that were differentially expressed were validated by qRT-PCR (S1C Fig). We compared our data to published transcriptional profiles and found a significant overlap between genes upregulated in untreated *flcn-1(ok975)* animals versus genes upregulated in wt animals treated with NaCl or osmotic stress resistant strains including *osm-7* and *osm-11* [8] (S1D, S1E and S1F Fig and S2, S3 and S4 Tables). Altogether, these data suggest that *flcn-1* is involved in a mechanism of regulating the resistance to hyperosmotic stress.

### Loss of *flcn-1* increases glycogen content, which mediates resistance to hyperosmotic stress

**Fig 2:**
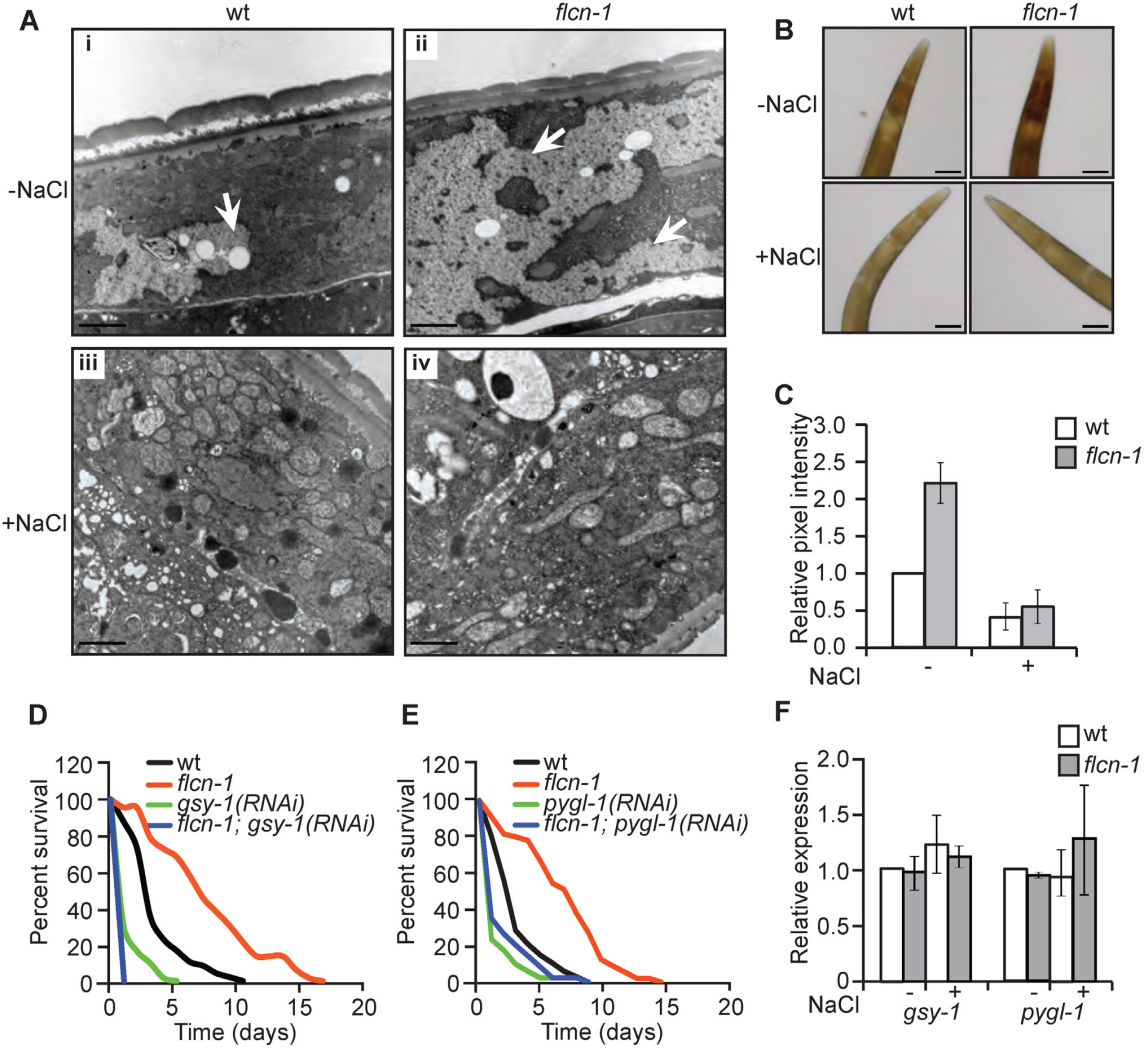
Loss of *flcn-1* increases glycogen content, which mediates resistance to hyperosmotic stress. (A) Representative electron micrographs from longitudinal sections of the hypodermis in indicated nematodes strains exposed or not to 400mM NaCl for 16 hours. Arrows represent glycogen stores. Scale bars: 2μm. (B, C) Iodine staining (B) and quantification of staining intensities (C) of indicated worm strains treated or not with 400mM NaCl for 16 hours. Data represent mean ± SEM, n≥ 3. (D, E) Percent survival to 400mM NaCl of indicated worm strains treated with indicated RNAi. (F) Relative mRNA levels of indicated target genes in indicated strains with or without 400mM NaCl treatment for 2 hours. Data represent the mean ± SEM, n≥ 3.

To determine how loss of *flcn-1* increases resistance to hyperosmotic stress, we assessed the morphological differences between wt and *flcn-1(ok975)* using electron microscopy with or without NaCl treatment. Interestingly, we observed an increase in the size and number of glycogen stores in adult (Fig 2Ai and 2Aii) and L4 (S2Ai, S2Aii, S2Ci, and S2Cii Fig) *flcn-1(ok975)* mutant worms as compared to wt. Specifically, our transmission electron data indicate a strong accumulation of glycogen in the hypodermis, muscle, and intestine of *flcn-1(ok975)* animals as compared to wt (S2C Fig). Glycogen has been previously shown to accumulate in these tissues in *C*. *elegans* [31]. Importantly, glycogen stores were barely detectable in wt and *flcn-1(ok975)* animals after NaCl treatment, suggesting that glycogen degradation is used to protect the animals from hyperosmotic stress (Fig 2Aiii, 2Aiv). Furthermore, we found that the prominent accumulation and salt stress-dependent degradation of glycogen in *flcn-1(ok975)* adult animals occurs in the hypodermis (Figs 2A, S2A and S2C). We validated and quantified the increase in glycogen levels conferred by loss of *flcn-1* using iodine staining which has been previously shown to specifically stain glycogen in *C*. *elegans* [32–34] (Fig 2B and 2C). In accordance with the electron microscopy results, glycogen levels were significantly increased in untreated *flcn-1(ok975)* animals as compared to wt, and NaCl treatment severely reduced glycogen content in both wt and *flcn-1(ok975)* animals (Fig 2B and 2C).

We then asked whether glycogen is used to protect wt and *flcn-1(ok975)* animals from damage during hyperosmotic stress. Glycogen synthase (*gsy-1)* is responsible for the synthesis of glycogen from UDP-glucose molecules and glycogen phosphorylase (*pygl-1)* catalyzes glycogen breakdown to form glucose-1-phosphate [35]. Importantly, the inhibition of glycogen synthesis or degradation using RNAi against *gsy-1* and *pygl-1* respectively, strongly reduced the survival in both wt and *flcn-1(ok975)* animals to an equal level, suggesting that the accumulation of glycogen and its degradation are both required for the resistance of wt and *flcn-1(ok975)* mutant animals to hyperosmotic stress (Fig 2D and 2E and S1 Table).

Additionally, transcript levels of *gsy-1* and *pygl-1* with or without 2 hours of 400mM NaCl stress remained unchanged suggesting allosteric regulation of glycogen metabolism (Fig 2F). Altogether, these results demonstrate that the accumulation of glycogen stores and the degradation of glycogen are essential to survive hyperosmotic stress in wt and *flcn-1(ok975)* mutant animals.

### Hyperosmotic stress resistance of *flcn-1(ok975)* animals is dependent on AMPK

**Fig 3:**
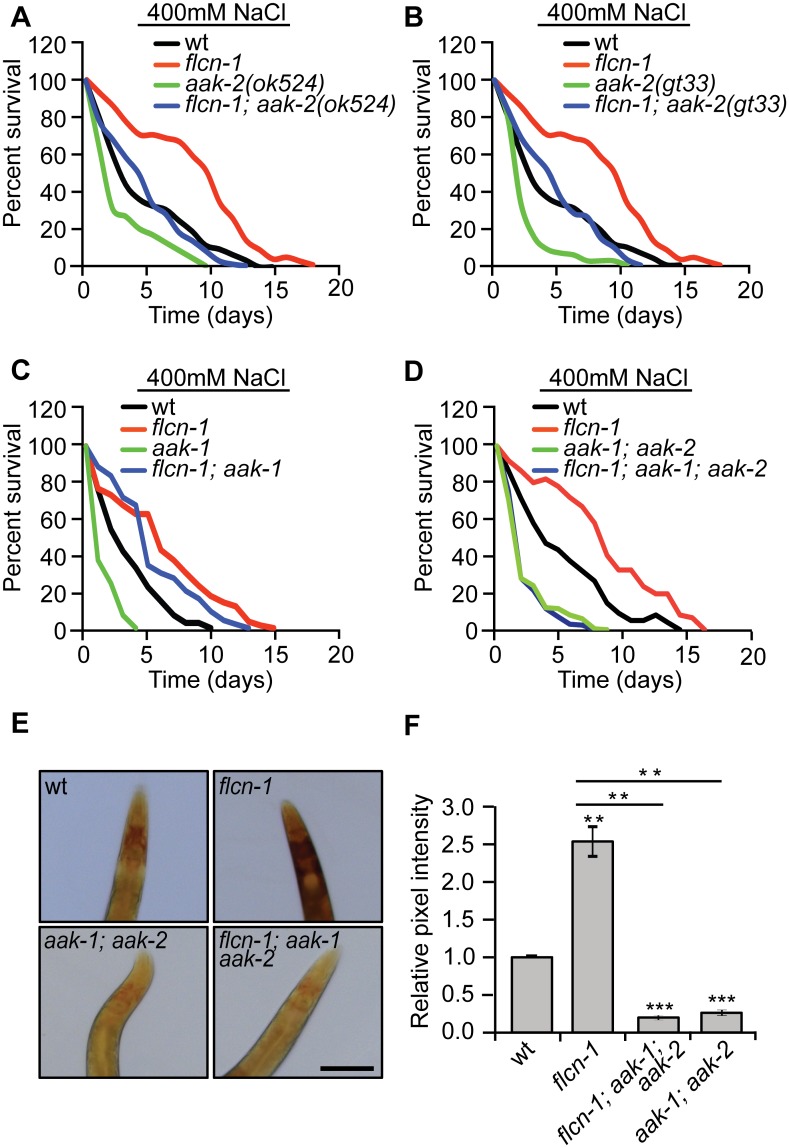
The increased survival to hyperosmotic stress and the accumulation of glycogen in *flcn-1* mutant worms require AMPK. (A-D) Percent survival of indicated worm strains exposed to 400mM NaCl. (A) *aak-2(ok524)*, (B) *aak-2(gt33)*, (C) *aak-1(tm1944)*, (D) *aak-1(tm1944)*; *aak-2(ok524)*. (E, F) Iodine staining (E) and quantification of staining intensities (F) of indicated worm strains. Scale bar:100μm. Data represent the mean ± SEM of at least 3 independent experiments.

Since we have previously reported that the *flcn-1*-dependent resistance to energy stresses requires *aak-2*, the worm ortholog of the AMPKα subunit, we wondered whether the hyperosmotic stress resistance phenotype conferred by loss of *flcn-1* is also mediated by AMPK [28]. AMPK is activated by hyperosmotic stress in mammalian systems [36] and its deletion confers sensitivity to NaCl stress in yeast [37]. *C*. *elegans* nematodes have two catalytic α subunits *aak-1* and *aak-2*. Loss of *aak-2* was shown to mediate lifespan extension and resistance to various stresses including oxidative stress, anoxia, nutrient deprivation, and dietary restriction [38–42]. To determine whether AMPK is involved in the increased resistance of *flcn-1(ok975)* animals to stress, we crossed *aak-2(ok524 and gt33)* [39,43] and *aak-1(tm1944)* [43] loss of function mutants with *flcn-1(ok975)* animals. Interestingly, loss of *aak-2(ok524* and *gt33)* or *aak-1(tm1044)* alone conferred stress sensitivity but did not fully suppress the increased survival to hyperosmotic stress conferred by loss of *flcn-1* (Fig 3A, 3B and 3C and S1 Table). To control for compensatory effects, we generated the *flcn-1(ok975); aak-1(tm1944); aak-2(ok524)* triple mutant and compared its survival under high salt conditions to *aak-1(tm1944); aak-2(ok524)* double mutant animals. Simultaneous loss of *aak-1* and *aak-2* completely abolished the increased osmotic stress resistance upon loss *flcn-1* demonstrating that this phenotype requires both AMPK catalytic subunits (Fig 3D and S1 Table).

### The accumulation of glycogen in *flcn-1* mutant worms depends on AMPK

AMPK has been shown to regulate glycogen metabolism in different organisms [44–56]. In fact, acute activation of AMPK leads to glycogen degradation [44–47], while chronic AMPK activation results in glycogen accumulation [48–50]. Since we observed an increased constitutive phosphorylation of AMPK upon loss of *flcn-1* in nematodes and mammalian cells [28,29], we hypothesized that the chronic AMPK activation in *flcn-1(ok975)* mutants may lead to increased glycogen levels. We determined glycogen levels in *aak-1(tm1944); aak-2(ok524)* animals compared to *flcn-1(ok975); aak-1(tm1944); aak-2(ok524)* triple mutant worms and found that loss of AMPK strongly reduced glycogen levels in both strains (Fig 3E and 3F). This suggests that the chronic AMPK activation in *flcn-1* animals is leading to glycogen accumulation. Interestingly, the survival and glycogen accumulation in *aak-1(tm1944); aak-2(ok524)* mutant animals was also severely reduced as compared to wt (Fig 3E and 3F), suggesting an important role for AMPK in maintaining glycogen stores, which are used for hyperosmotic stress resistance.

### Autophagy is not fully required for the hyperosmotic stress resistance conferred by loss of *flcn-1*

Autophagy is a biological survival process through which cellular components and damaged organelles are degraded to produce energy upon starvation [57]. We reported previously that autophagy was essential for the energy stress resistance of *flcn-1(ok975)* mutant animals [28]. Therefore, we asked whether autophagy plays a role in osmotic stress resistance. Interestingly, *atg-18(gk378)* mutant animals were hypersensitive to high salt concentrations suggesting that autophagy is a process involved in the resistance to hyperosmotic stress. However, loss of *flcn-1* significantly increased the resistance of *atg-18(gk378)* animals suggesting that *flcn-1*-dependent hyperosmotic stress resistance does not require autophagy, which is different from what we observed before during energy stress [28] (S3 Fig and S1 Table).

### Glycogen degradation leads to heightened glycerol levels and protects animals from hyperosmotic stress

**Fig 4:**
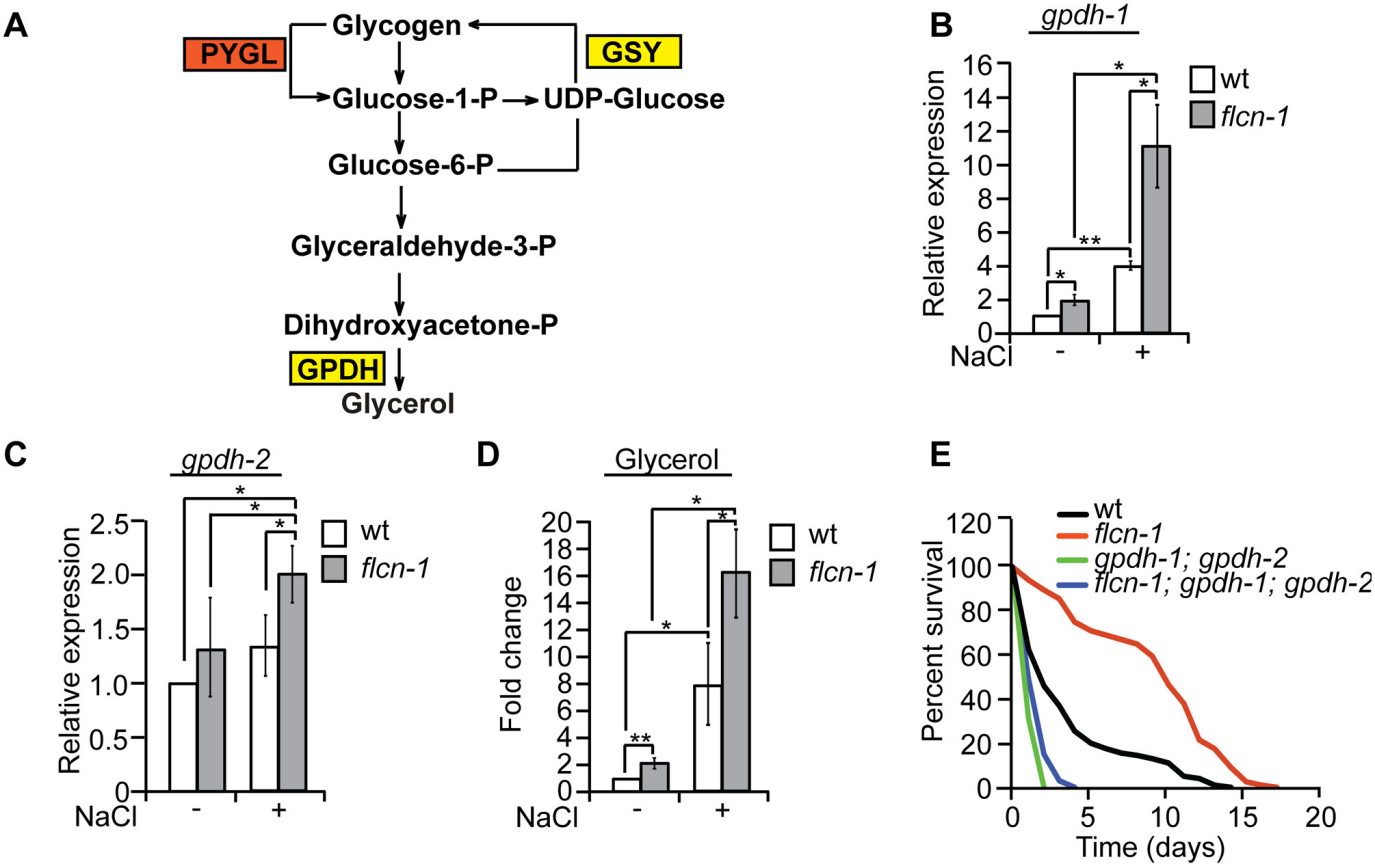
Glycogen degradation heightens glycerol levels and protects animals from hyperosmotic stress. (A) Representative scheme of glycogen metabolism and osmolyte production in worms. (B-D) Relative mRNA levels of *gpdh-1* and *gpdh-2* (B, C) and glycerol content (D) in wt and *flcn-1(ok975)* L4/young adult animals treated with or without 400mM NaCl for 2 hours. Data represent mean ± SEM, n ≥3. (E) Percent survival of indicated worm strains exposed to 400mM NaCl.

Degradation of glycogen polymers leads to the formation of glucose-1-phosphate which is converted to glucose-6-phosphate, an important metabolite used in multiple pathways including glycolysis, pentose phosphate pathway, and glycerol production (Fig 4A) [35]. We hypothesized that glycogen degradation may lead to heightened glycerol levels that could protect the animals from hyperosmotic stress. To address this, we measured the mRNA levels of *gpdh-1* and *gpdh-2*. Interestingly, we observed a significant 2-fold increase in *gpdh-1* but not *gpdh-2* at unstressed conditions in *flcn-1(ok975)* mutant animals compared to wt, which was consistent with our microarray results (Fig 4B and 4C and S2 Table). Strikingly, after 2 hour treatment with 400mM NaCl, we detected a strong induction of *gpdh-1* and *gpdh-2* mRNA levels in wt and *flcn-1(ok975)* mutant animals, which was significantly enhanced in the latter (Fig 4B and 4C). Accordingly, *flcn-1(ok975)* mutant animals exhibit higher glycerol content at basal level as compared to wt animals which was further increased upon NaCl treatment (Fig 4D).

To determine the importance of glycerol in the protection against hyperosmotic stress, we inhibited *gpdh-1* and *gpdh-2* using RNAi and using mutant strains. Importantly, treatment of *flcn*-*1(ok975)* animals with RNAi against either *gpdh-1* or *gpdh-2* alone did not fully suppress the increased resistance of *flcn-1(ok975)* animals to hyperosmotic stress (S4A and S4B Fig). We then compared the resistance of *flcn-1(ok975); gpdh-1(kb24); gpdh-2(kb33)* triple mutant animals to *gpdh-1(kb24); gpdh-2(kb33)* mutant nematodes. Simultaneous loss of *gpdh-1* and *gpdh-2* strongly reduced the survival of *flcn-1(ok975)* mutant animals demonstrating an important role for the osmolyte glycerol in the survival of *flcn-1(ok975)* and wt animals (Fig 4E and S1 Table). Altogether, these data suggest that upon hyperosmotic stress glycogen stores are metabolized into the osmolyte glycerol via enhanced transcriptional upregulation of *gpdh* enzymes. This glycerol mediated osmo-protective phenotype is significantly enhanced upon loss of *flcn-1* in nematodes.

### Loss of *pmk-1* does not fully suppress the hyperosmotic stress resistance conferred by loss of *flcn-1*

HOG/p38/PMK-1 MAP kinase signaling is widely known to control adaptation to hypertonic stresses in multiple organisms [4,9,58]. As expected, *pmk-1(km25)* mutant worms were highly sensitive to osmotic stress. However, loss of *pmk-1* in *flcn-1(ok975)* mutant animals reduced but did not fully suppress the increased resistance conferred by *flcn-1* depletion (S5A Fig and S1 Table). Supporting this result, the expression of *gpdh-1* is ~2-fold higher in *flcn-1(ok975); pmk-1(km25)* mutant animals as compared to *pmk-1(km25)* alone (S5B Fig). Altogether, this suggests that *pmk-1* is not involved in the transcriptional upregulation of *gpdh-1* upon loss of *flcn-1* and that it acts in parallel to *flcn-1* and *aak-1/2*.

### The increased accumulation of glycogen content conferred by loss of FLCN is conserved from *C*. *elegans* to humans

**Fig 5:**
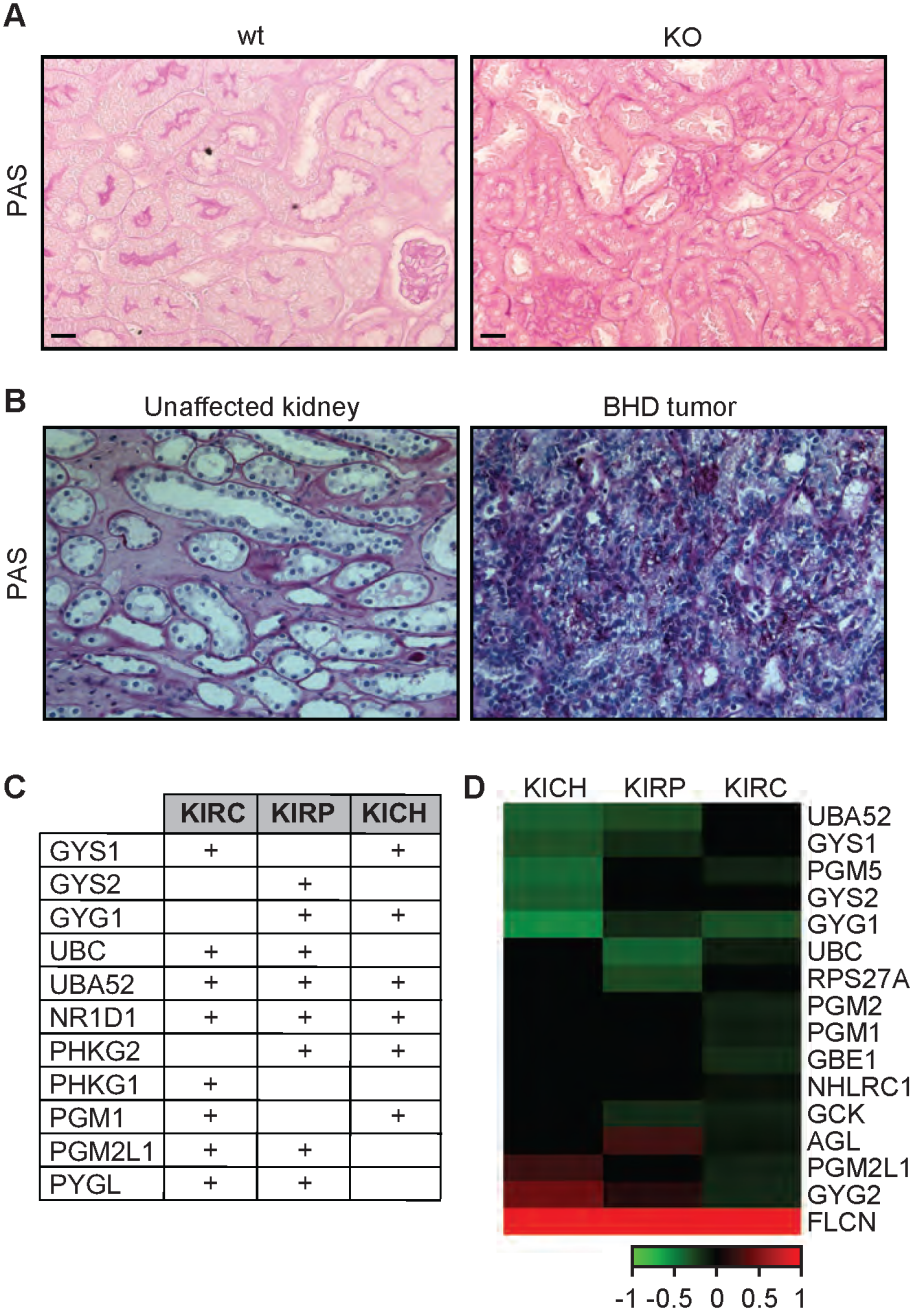
The FLCN-dependent glycogen accumulation is conserved from *C*. *elegans* to humans. (A-B) PAS staining of kidney sections from wt and *Flcn* kidney-specific KO mice (A) and human BHD kidney tumor in comparison with an adjacent region from the same individual (B). Scale bars:100μm. (C) Table indicating the upregulated glycogen metabolism genes in kidney tumors (KIRC, KIRP, and KICH) as compared to normal kidney. The sign (+) indicates genes that are upregulated in these tumors. The values are indicated in S5 Table. (D) Heat map indicating correlation of expression between glycogen metabolism genes and *FLCN* in KIRC, KIRP, and KICH tumors. Green and red colors indicate genes that are negatively or positively correlated with FLCN expression, respectively.

Glycogen is linked to the progression and the aggressiveness of multiple cancer types in humans [59,60]. To determine whether loss of FLCN also leads to the accumulation of glycogen in mammalian systems, we used the *Flcn* ^*flox/flox*^/*Pax8-Cre* mouse model where *Flcn* is specifically deleted in the kidney and determined glycogen content using Periodic-Acid-Schiff (PAS) staining. The *Flcn* ^*flox/flox*^/*Pax8-Cre* mouse was generated by mating *Pax8-Cre* mice with the *Flcn* ^*flox/flox*^ C57BL/6 mice. By six months of age, all mice developed visible macroscopic lesions confirmed as cysts that later developed into tumors. Strikingly, kidneys from *Flcn* ^*flox/flox*^/*Pax8-Cre* mice accumulated higher glycogen levels as compared to normal kidneys from *Flcn* ^*flox/flox*^ mouse littermates (Figs 5A and S6A). Our data show a stronger glycogen accumulation in the kidney cortex, which is due to the fact that *Pax8* is expressed in the epithelial cells of the proximal and distal renal tubules, loops of Henle, collecting ducts and the parietal epithelial cells of Bowman’s capsule [61]. Importantly, PAS staining of tumors from BHD patients also indicate a strong accumulation of glycogen as compared to adjacent unaffected kidneys (Figs 5B and S6B). We also compared the expression level of glycogen biosynthesis and degradation genes in 3 different subtypes of kidney cancer, kidney renal papillary cell carcinoma (KIRP), kidney renal clear cell carcinoma (KIRC), and kidney chromophobe (KICH) tumors. Strikingly, we observed a significant upregulation of genes involved in the synthesis and degradation of glycogen (Fig 5C and S5 Table). We also observed that the expression of 46% of these genes are negatively correlated with FLCN expression (Fig 5D). Overall, our data indicate that the accumulation of glycogen upon loss of FLCN is be conserved from nematodes to mammals, and that it might play a role in tumorigenesis.

## Discussion

**Fig 6:**
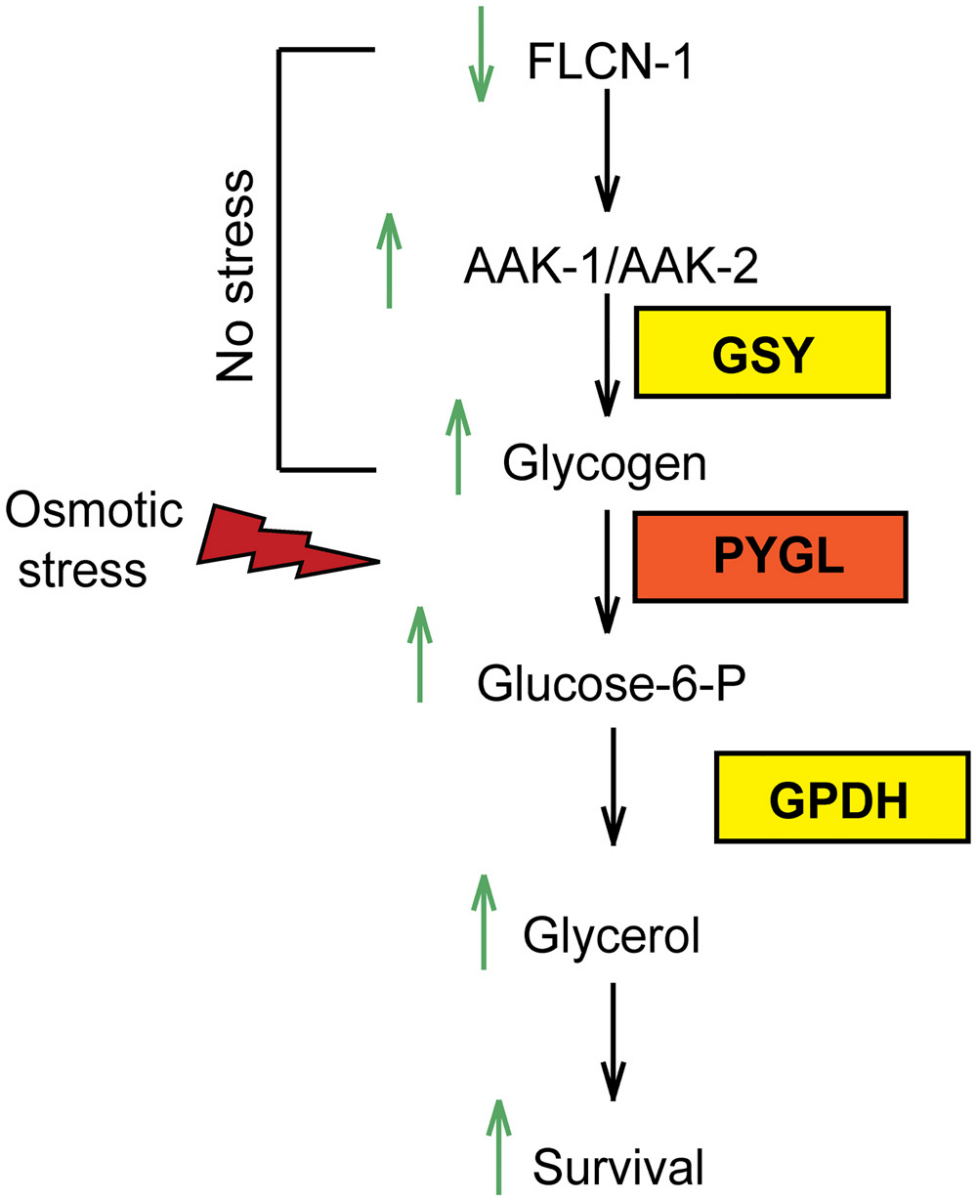
Graphical representation of FLCN-1/AMPK hyperosmotic stress resistance pathway. Loss of *flcn-1* chronically activates AMPK and leads to glycogen accumulation under normal conditions. Upon exposure to hyperosmotic stress, glycogen is rapidly degraded leading to the production of glycerol and animal survival.

A common mechanism to survive osmotic stress is the synthesis of compatible osmolytes [3]. In yeast and in *C*. *elegans*, the rapid accumulation of glycerol after hyperosmotic stress has been demonstrated [4,5]. However, it is not clear what fuels glycerol production upon acute hyperosmotic stress. Here we show that animals have evolved an interesting strategy to maintain glycogen stores, which can serve as fuel for glycerol production to ensure survival to acute hyperosmotic stress (Fig 6). While storage of soluble glucose molecules in cells would lead to osmotic stress, the storage of glucose in the form of insoluble glycogen polymers ensures osmotic homeostasis. Importantly, our data uncover that glycogen stores have a dual role: they can serve as a reservoir for production of energy or osmolytes. Indeed, pretreatment of wt and *flcn-1(ok975)* animals with oxidative and energy stressor paraquat, depletes glycogen stores rapidly and suppresses survival upon treatment with 400mM NaCl (S2A and S2B Fig).

The regulation of glycogen metabolism by AMPK has long been a paradox [44–50]. Acute activation of AMPK, by in vitro short term treatment of the AMP mimetic drug 5-Aminoimidazole-4-Carboxamide Riboside (AICAR), leads to the phosphorylation and inhibition of glycogen synthase, which favors glycogen degradation for supply of short term energy [44–47]. However, chronic AMPK activation induced by a long term AICAR treatment or by genetic manipulation of AMPK regulatory subunits, results in glycogen accumulation via glucose-6-phosphate-dependent allosteric activation of glycogen synthase, which bypasses the inhibitory effect of the AMPK-mediated phosphorylation [48–50]. In agreement, constitutive AMPK activation through transgenic expression of activating mutations in the γ2 and γ3 subunits in mice and pigs leads to substantial glycogen accumulation in cardiac and skeletal muscles [36,50–53,55,56]. In light of these results, our data indicate that chronic AMPK activation upon loss of *flcn-1* leads to glycogen accumulation. Similarly to what has been shown in yeast [54], we demonstrate that AMPK-deficient strains exhibit reduced glycogen content as compared to wt. We further show that the accumulation of glycogen in wt and *flcn-1(ok975)* mutant animals depends on AMPK. Based on the data presented here together with our recently published reports [28,29], we propose that FLCN is a key regulatory component of AMPK.

*Flcn* muscle-specific knockout mice and *Fnip1* knockout mice exhibited increased glycogen accumulation in muscles and liver, respectively [62,63]. Here we show that loss of FLCN leads to glycogen accumulation in kidneys of mice and in the tumors of BHD patients, suggesting that this pathway is evolutionarily conserved. In agreement with the important role for glycogen in organismal survival to stress, glycogen can be used by tumor cells to survive harsh microenvironments such as hypoxia [59,64]. In fact, glycogen accumulates in many cancer types [64] and inhibition of its degradation led to induction of apoptosis and impaired *in vivo* growth of tumor xenografts [59].

Importantly, our data might impinge on a novel role for glycogen in tumorigenesis. In addition to its critical role as an energy supplier, we speculate that glycogen degradation might lead to higher osmolyte levels to help survive hyperosmotic tumor microenvironments. In fact, we found that taurine and sorbitol synthesis genes, CSAD and AKR1B1 respectively, are upregulated in many kidney tumors (S5 Table). Supporting this idea, recent evidence shed light on an important role of the nuclear factor of activated T cells 5 (NFAT5), a major transcription factor that regulates osmotic stress resistance genes, in promoting tumorigenesis and metastasis of several cancer types [2,65–67]. In summary, we speculate that the increased glycogen stores in tumors might lead to extended survival of cells under hyperosmotic stress, which could ultimately lead to neoplastic transformation by accumulation of DNA damage [1, 2].

## Materials and Methods

### *C*. *elegans* strains, maintenance, and RNAi treatments

*C*. *elegans* strains were obtained from the *Caenorhabditis* Genetics Center (S6 Table). Nematodes were maintained and synchronized using standard culture methods [68]. The RNAi feeding experiments were performed as described in [69], and bacteria transformed with empty vector were used as control. For all RNAi experiments, phenotypes were scored with the F1 generation.

### Osmotic stress resistance assay

To measure osmotic stress resistance, synchronized 1 day adult worms were transferred to high concentration NaCl plates. Survival was measured daily. Worms that responded by movement to touch with the platinum wire were considered as alive.

### Percent recovery assay

To measure the percentage of animals that recovered after hyperosmotic shock, 1 day adult animals were transferred to high NaCl plates. Animals shrink and paralyse shortly after exposure to NaCl. After 2 hours, animals that were able to move their entire body forward or backward in response to touch with a platinum wire were considered as “recovered”. Paralyzed animals often look straight and are unable to move.

### RNA extraction and real-time PCR

Synchronized young adult nematodes were harvested and total RNA was extracted with Trizol. Reverse transcription and qRT-PCRs were performed as previously described [28]. Transcripts were normalized to *cdc-42*.

### Microarray experiment and gene overlap analysis

Synchronized young adult wt and *flcn-1(ok975)* animals were harvested and RNA was extracted using Trizol and purified using Qiagen RNeasy columns. Total RNA samples were then hybridized onto Agilent gene chips. Fold change values are calculated using the mean of both data sets. The overlapping genes between *flcn-1(ok975)* mutant animals and the specified conditions and strains [8] were performed using the “compare two lists” online tool at http://www.nemates.org/MA/progs/Compare.html. The significance of the overlap and enrichment scores were determined via hypergeometric distribution method using http://nemates.org/MA/progs/overlap_stats.html. The number of genes in the *C*. *elegans* genome was considered to be19,735.

### Transmission electron microscopy

Synchronized 1 day adult nematodes were transferred to 400mM NaCl plates for 16 hours. Recovering animals were picked and transferred for TEM. Immersion fixation and embedding was performed according to [70]. Thin sections were cut on an RMC Powertome XL (Boeckler Instruments) using a diamond knife (DDK) and collected on Pioloform-coated copper slot grids. Grids were post-stained with 4% uranyl acetate and lead citrate and viewed using a Philips CM10 electron microscope (FEI) equipped with a Morada digital camera (Olympus) and iTEM software (Olympus SIS).

### Glycogen quantification in *C*. *elegans*

Synchronized young adult animals were transferred to agarose pads. For comparisons between strains, different conditions were transferred to the same agarose pad and were exposed to iodine vapor for 30 seconds. Animals were rapidly imaged individually. Quantification of the intensity of the staining was performed using ImageJ software.

### Periodic acid Schiff staining

For human normal kidney and BHD tumor samples, slides were rehydrated after deparaffination and treated with 1% periodic acid for 10 minutes. Periodic acid was washed off with H_2_O and slides were then incubated in Schiff reagent for 20 min. Slides were then rinsed with H_2_O, counterstained with hematoxylin and embedded in entellan. Images were taken as described in [71].

### Glycerol determination in *C*. *elegans*

Synchronized L4/young adult animals exposed or not to 400mM NaCl for 2 hours and were harvested and washed with M9 buffer adjusted to match plate salinity. Pellets were flash frozen in liquid nitrogen. Extraction was performed according to [5]. Briefly, frozen pellets were ground using a cold mortar and pestle on dry ice. The worm powder was then resuspended in 1N perchloric acid, and solutions were transferred to 15ml conical tubes and kept on ice for 1 hour. The lysate was then centrifuged and the supernatant was neutralized with 5N KOH containing 61.5mM K_2_HPO_4_ and 38.5mM KH_2_PO_4_. Glycerol levels were determined using a glycerol determination kit (R-Biopharm, Marshall, MI). Pellets were solubilized in 0.1N NaOH and protein content was determined using BCA. Glycerol levels were normalized to protein content.

### Gene expression analysis in kidney cancers from patients

TCGA data including 91 kidney chromophobe gene expression RNASeq (IlluminaHiSeq), 604 kidney renal clear cell carcinoma gene expression RNASeq (IlluminaHiSeq), and 258 kidney renal papillary cell carcinoma gene expression RNASeq (IlluminaHiSeq), were extracted from cancer Genomics Browser (https://genome-cancer.ucsc.edu/proj/site/hgHeatmap). For expression analysis, data were expressed as median fold change and the Mann-Whitney test was used to calculate the p-values between normal and tumor samples. P-values less than 0.05 were considered to be statistically significant. For correlation analysis TCGA expression data (same as expression analysis) were used to calculate the Pearson correlation coefficient, and generate a heat map, using R software 3.1.1 (http://www.r-project.org/). P-values less than 0.05 were considered to be statistically significant.

### Statistical analyses

Data are expressed as means ±SEM. Statistical analyses for all data were performed by student's t-test, using Excel (Microsoft, Albuquerque, NM, USA). For hyperosmotic stress survival curve comparisons we used the Log-rank Mantel Cox test using GraphPad software. Statistical significance is indicated in figures (* *P<*0.05, **P<0.01, ***P<0.001) or included in the supplemental tables.

## Supporting Information

S1 Fig Transcriptional profile of *flcn-1* prior to stress overlap with profiles of wt animals exposed to NaCl and to osmotic stress resistant mutants. (A) Western blot showing the expression of FLCN-1 in indicated strains. (B) Lifespan of wt and *flcn-1(ok975)* animals at 20°C. (C) Relative mRNA levels of indicated target genes in wt and *flcn-1(ok975)* animals. (D-F) Ven diagrams showing the overlap of genes in indicated strains and treatments.
(EPS)

S2 Fig Pretreatment of wt and *flcn-1(ok95)* animals with paraquat suppresses hyperosmotic stress resistance. (A) Electron micrographs showing glycogen stores in wt and *flcn-1(ok975)* L4/young adult animals with or without 50mM paraquat treatment for 2 hours. Scale bars: 0.5 μm. (B) Percent survival of indicated worm strains pretreated with 70mM PQ for 5 hours followed by exposure to 400mM NaCl. (C) Electron micrographs showing glycogen stores in cross sections from wt and *flcn-1(ok975)* L4/young adult animals (i, ii), and in the head region of a *flcn-1(ok975)* adult animal (iii). H: hypodermis. M: muscle. I: intestine. Scale bars: 5 μm (i, ii) and 2 μm (iii). Arrows indicate glycogen stores.
(EPS)

S3 Fig The increased resistance of *flcn-1(ok975)* animals to NaCl does not fully require autophagy. Percent survival of indicated worm strains exposed to 400mM NaCl.
(EPS)

S4 Fig GPDH-1 is critical for the survival of *flcn-1(ok975)* animals to hyperosmotic stress. (A-B) Percent survival of wt and *flcn-1(ok975)* mutant animals treated with indicated RNAi.
(EPS)

S5 Fig Involvement of PMK-1 in the transcriptional response of *gpdh-1* and response to hyperosmotic stress. (A) Percent survival of indicated worm strains exposed to 400mM NaCl, *pmk-1(km25)*. (B) Relative mRNA levels of *gpdh-1* in indicated worm strains.
(EPS)

S6 Fig The FLCN-dependent glycogen accumulation is conserved from *C*. *elegans* to mammals. (A-B) Microscopy images showing PAS and H&E staining of kidney sections from wt and *Flcn* kidney-specific KO mice (A) and a human BHD tumor (B). Four individual images were merged in panel B. Scale bars: 200μm.
(EPS)

S1 Table Mean survival on NaCl plates: results and statistical analysis. (DOCX)

S2 Table Overlapping genes upregulated in *flcn-1(ok975)* animals at basal level and wild-type animals treated with NaCl. (DOCX)

S3 Table Overlapping genes upregulated in *flcn-1(ok975)* animals and *osm-7(n1515)* animals. (DOCX)

S4 Table Overlapping genes upregulated in *flcn-1(ok975)* animals and *osm-11(n1604)* animals. (DOCX)

S5 Table Glycogen metabolism gene regulation in KIRC, KIRP and KICH kidney tumors. (DOCX)

S6 Table Strains list. (DOCX)
