# Supplementary material for: FLCN and AMPK Confer Resistance to Hyperosmotic Stress via Remodeling of Glycogen Stores
Source: PLoS Genet. 2015 Oct 6;11(10):e1005520. doi: 10.1371/journal.pgen.1005520 (PMC4595296; doi:10.1371/journal.pgen.1005520)
Supplement: S1 Table — (DOCX) [file pgen.1005520.s007.docx]

| **Table S1: Mean survival on NaCl plates^a^: results and statistical analysis^a^** | | | |
| --- | --- | --- | --- |
| Strain, RNAi | Mean survival (days ± SEM) | p-value | Number of experiments  (n) |
| N2 (untreated) | 14.1 ± 1.62 |  | 3 |
| *flcn-1(ok975)* (untreated) | 13.7 ± 1.94 | n.s.^b^ | 3 |
| N2 | 4.79 ± 1.11 |  | 21 |
| *flcn-1(ok975)* | 7.89 ± 1.70 | <0.0001^c^ | 21 |
| N2 (500mM NaCl) | 2.58 ± 1.38 |  | 7 |
| *flcn-1(ok975)* (500mM NaCl) | 6.46 ± 1.03 | <0.0001^d^ | 7 |
| *flcn-1(ok975)* | 7.15 ± 0.83 |  | 3 |
| *flcn-1(ok975); flcn-1:GFP MA2* | 4.60 ± 1.17 | <0.0001^e^ | 3 |
| *aak-2(ok524)* | 2.37 ± 0.77 | <0.0001^f^ | 3 |
| *flcn-1(ok975); aak-2(ok524)* | 3.81 ± 1.05 | <0.0001^g, h^ | 3 |
| *aak-1(tm144)* | 1.79 ± 0.09 | <0.0001^i^ | 3 |
| *flcn-1(ok975); aak1(tm1944)* | 5.20 ± 0.38 | <0.0001^j, k^ | 3 |
| *aak-1(tm1944);aak-2(ok524)* | 2.64 ± 0.64 | <0.0001^l^ | 5 |
| *flcn-1(ok975);aak-1(tm1944);aak-2(ok524)* | 2.51 ± 0.60 | <0.0001^m^/n.s. ^n^ | 5 |
| *atg-18(gk378)* | 1.40 ± 0.24 | <0.0001^o^ | 4 |
| *flcn-1(ok975); atg-18(gk378)* | 3.12 ± 0.50 | <0.0001^p, q^ | 4 |
| *aak-2(gt33)* | 2.14 ± 0.54 | <0.0001^r^ | 3 |
| *flcn-1(ok975); aak-2(gt33)* | 4.36 ± 0.64 | <0.0001^s, t^ | 3 |
| *pmk-1(km25)* | 1.34 ± 0.26 | <0.0001^u^ | 5 |
| *flcn-1 (ok975)*; *pmk-1(km25)* | 2.43 ± 0.23 | <0.0001^v, w^ | 5 |
| *gpdh-1(kb24); gpdh-2(kb33)* | 1.25 ± 0.09 | <0.0001^x^ | 3 |
| *flcn-1; gpdh-1(kb24); gpdh-2(kb33)* | 1.59 ± 0.16 | <0.0001^y, z^ | 3 |
| N2 (*ev*) | 3.24 ± 1.03 |  | 7 |
| *flcn-1 (ev)* | 5.74 ± 1.74 | <0.0001^aa^/0.0069^bb^ | 7 |
| N2 (*gsy-1* RNAi) | 1.20 ± 0.19 | <0.0001^cc^ | 4 |
| *flcn-1(ok975)*(*gsy-1* RNAi) | 1.22 ± 0.22 | <0.0001^dd^/n.s. ^ee^ | 4 |
| N2 (*gpdh-1* RNAi) | 1.64 ± 0.09 | <0.0001^ff^ | 3 |
| *flcn-1(ok975)*(*gpdh-1* RNAi) | 2.68 ± 1.03 | <0.0001^gg,^ ^hh^ | 3 |
| N2 *(pygl-1* RNAi*)* | 1.37 ± 0.37 | <0.0001^ii^ | 4 |
| *flcn-1(ok975)*(*pygl-1* RNAi*)* | 1.47 ± 0.37 | <0.0001^jj^/n.s. ^kk^ | 4 |
| N2 (*gpdh-2* RNAi) | 3.43 ± 1.08 | <0.0001^ll^ | 3 |
| *flcn-1(ok975)*(*gpdh-2* RNAi) | 7.42 ± 0.07 | <0.0001^mm,^ ^nn^ | 3 |

1. Survival on 400mM NaCl and Mantel-Cox statistics on pooled results have been performed unless noted otherwise
2. Compared to N2 animals
3. Compared to N2 animals
4. Compared to *N2* animals
5. Compared to *flcn-1(ok975)* animals
6. Compared to N2 animals
7. Compared to *aak-2 (ok524)* animals
8. Compared to *flcn-1(ok975)* animals
9. Compared to N2 animals
10. Compared to *aak-1 (tm1944)* animals
11. Compared to *flcn-1(ok975)* animals
12. Compared to N2 animals
13. Compared to *flcn-1(ok975)* animals
14. Compared to *aak-1(tm1944); aak-2(ok524)* animals
15. Compared to N2 animals
16. Compared to *atg-18 (gk378)* animals
17. Compared to *flcn-1(ok975)* animals
18. Compared to N2 animals
19. Compared to  *aak-2(gt33)* animals
20. Compared to *flcn-1(ok975)* animals
21. Compared to N2 animals
22. Compared to *pmk-1(ku25)* animals
23. Compared to *flcn-1(ok975)* animals
24. Compared to N2 animals
25. Compared to *flcn-1(ok975)* animals
26. Compared to *gpdh-1(kb24); gpdh-2(kb33)* animals
27. Compared to N2 animals (Mantel-Cox)
28. Compared to N2 animals (t-test for means)
29. Compared to N2 animals treated with control RNAi
30. Compared to *flcn-1(ok975)* animals treated with control RNAi
31. Compared to N2 animals treated with *gsy-1* RNAi
32. Compared to N2 animals treated with control RNAi
33. Compared to *flcn-1(ok975)* animals treated with control RNAi
34. Compared to N2 animals treated with *gpdh-1* RNAi
35. Compared to N2 animals treated with control RNAi
36. Compared to *flcn-1(ok975)* animals treated with control RNAi
37. Compared to N2 animals treated with *pygl-1* RNAi
38. Compared to N2 animals treated with control RNAi
39. Compared to *flcn-1(ok975)* animals treated with control RNAi
40. Compared to N2 animals treated with *gpdh-2* RNAi
