# Supplementary material for: FLCN and AMPK Confer Resistance to Hyperosmotic Stress via Remodeling of Glycogen Stores
Source: PLoS Genet. 2015 Oct 6;11(10):e1005520. doi: 10.1371/journal.pgen.1005520 (PMC4595296; doi:10.1371/journal.pgen.1005520)
Supplement: S2 Table — (DOCX) [file pgen.1005520.s008.docx]

| **Table S2: Overlapping genes upregulated in *flcn-1(ok975)* animals at basal level and wild-type animals treated with NaCl** | | | | | |
| --- | --- | --- | --- | --- | --- |
| **Gene** | **Gene ID** | **Sequence description** | **Fold change** | **P value** | **ES** |
| C31A11.5 | *oac-6* | O-acyltransferase homolog | 2.862762 | 0.00026 | 3.0 |
| C49G7.10 | C49G7.10 |  | 1.642492 | 0.0030 |  |
| F10F2.2 | F10F2.2 | Ortholog of human phosphoribosylformylglycinamidine synthase | 1.50 | 0.00015 |  |
| F13A7.11 | F13A7.11 |  | 1.980989 | 0.0021 |  |
| F15B9.1 | *far-3* | Fatty Acid/Retinol binding protein | 1.399218 | 0.0028 |  |
| F15E11.12 | F15E11.12 |  | 2.193222 | 6.29E-07 |  |
| F47G4.3 | *gpdh-1* | Glycerol -3- phosphate dehydrogenase 1 | 1.724423 | 1.47E-05 |  |
| F53A9.2 | F53A9.2 | Ortholog of human histidine-rich glycoprotein | 2.227965 | 4.74E-05 |  |
| F53B2.8 | F53B2.8 |  | 2.258014 | 0.00087 |  |
| H17B01.3 | *lips-14* | Lipase related | 1.797276 | 1.78E-05 |  |
| R09B5.9 | *cnc-4* | Caenanin family | 1.98787 | 2.08E-07 |  |
| T12D8.5 | T12D8.5 |  | 2.140247 | 0.00019 |  |
| T24C4.4 | T24C4.4 |  | 2.781296 | 0.0012 |  |
