# Supplementary material for: FLCN and AMPK Confer Resistance to Hyperosmotic Stress via Remodeling of Glycogen Stores
Source: PLoS Genet. 2015 Oct 6;11(10):e1005520. doi: 10.1371/journal.pgen.1005520 (PMC4595296; doi:10.1371/journal.pgen.1005520)
Supplement: S3 Table — (DOCX) [file pgen.1005520.s009.docx]

| **Table S3: Overlapping genes upregulated in *flcn-1(ok975)* animals and *osm-7(n1515)* animals** | | | | | |
| --- | --- | --- | --- | --- | --- |
| **Gene ID** | **Gene** | **Sequence description** | **Fold change** | **P value** | **ES** |
| B0213.14 | *cyp-34A8* | Cytochrome P450 family | 1.48 | 0.0017 | 6.1 |
| C14C6.5 | C14C6.5 | Contains a Methridin like shK toxin domain | 1.90 | 1.48E-06 |  |
| F01D5.5 | F01D5.5 |  | 1.507 | 0.0009 |  |
| F10F2.2 | F10F2.2 | Ortholog of human phosphoribosylformylglycinamidine synthase | 1.50 | 0.00015 |  |
| F15B9.1 | *far-3* | Fatty Acid/Retinol binding protein | 1.399218 | 0.0028 |  |
| F17E9.11 | *lys-10* | Lysozyme | 3.086142 | 0.00037 |  |
| F28D1.5 | *thn-2* | Thaumatin family | 2.728437 | 2.16E-11 |  |
| F41C3.1 | F41C3.1 |  | 2.029536 | 0.00306 |  |
| F47G4.3 | *gpdh-1* | Glycerol -3- phosphate dehydrogenase 1 | 1.724423 | 1.47E-05 |  |
| F53A9.1 | F53A9.1 | Ortholog of human histidine-rich glycoprotein | 1.553867 | 8.20E-05 |  |
| F55G11.4 | F55G11.4 | Contains a CUB-like domain | 2.53442 | 5.64E-09 |  |
| F59A7.2 | F59A7.2 |  | 1.621973 | 0.00067 |  |
| K08C7.5 | *fmo-2* | Flavin-containing Mono-oxygenase family | 3.01883 | 0.0011 |  |
| R09B5.9 | *cnc-4* | Caenacin family | 1.98787 | 2.08E-07 |  |
| T03D3.1 | T03D3.1 | UDP-GlucuronosylTransferase | 1.618075 | 0.0014 |  |
| T19B10.2 | *phi-59* |  | 1.640768 | 1.91E-05 |  |
| Y43C5A.2 | Y43C5A.2 |  | 1.374308 | 0.0030 |  |
| Y9C9A.16 | Y9C9A.16 | Ortholog of human sulfide quinone reductase-like | 2.773042 | 0.0001 |  |
| ZC204.12 | ZC204.12 |  | 1.58676 | 0.0014 |  |
| ZK970.7 | ZK970.7 |  | 1.414119 | 0.0007 |  |
