# Supplementary material for: FLCN and AMPK Confer Resistance to Hyperosmotic Stress via Remodeling of Glycogen Stores
Source: PLoS Genet. 2015 Oct 6;11(10):e1005520. doi: 10.1371/journal.pgen.1005520 (PMC4595296; doi:10.1371/journal.pgen.1005520)
Supplement: S5 Table — (DOCX) [file pgen.1005520.s011.docx]

| **Table S5: Glycogen metabolism gene regulation in KIRC, KIRP and KICH kidney tumors** | | | | | | | | | |
| --- | --- | --- | --- | --- | --- | --- | --- | --- | --- |
| **Genes** | **KICH** | | | **KIRC** | | | **KIRP** | | |
|  | **Median of tumor samples** | **Median of normal samples** | **P value** | **Median of tumor samples** | **Median of normal samples** | **P value** | **Median of tumor samples** | **Median of normal samples** | **P value** |
| **GYS1** | 0.2794 | -0.6122 | <0.0001 | 0.02552 | -0.7864 | <0.0001 | ns | ns | 0.84 |
| **GYS2** | ns^a^ | ns | 0.42 | ns | ns | 0.5 | 0.1384 | -0.6196 | <0.0001 |
| **GYG1** | 0.336 | -0.9145 | <0.0001 | ns | ns | 0.2 | 0.07869 | -0.1943 | <0.001 |
| **UBC** | 0.1075 | -0.8703 | 0.192 | 0.1075 | -0.8703 | <0.0001 | 0.02744 | -0.4162 | <0.0001 |
| **UBA52** | 0.0913 | -0.2527 | <0.001 | 0.063 | -0.5169 | <0.0001 | 0.04811 | -0.3756 | <0.0001 |
| **NR1D1** | 0.3206 | -0.8421 | <0.0001 | 0.157 | -0.8631 | <0.0001 | 0.2355 | -1.224 | <0.0001 |
| **PHKG2** | 0.153 | -0.3173 | <0.0001 | ns | ns | 0.34 | 0.04544 | -0.4429 | <0.0001 |
| **PHKG1** | ns | ns | 0.098 | 0.1104 | -1.118 | <0.0001 | ns | ns | 0.2 |
| **PGM1** | 0.1682 | -0.1728 | <0.001 | 0.0742 | -0.1665 | <0.0001 | 0.1336 | 0.07823 | 0.55 |
| **PGM2L1** | ns | ns | 0.5 | 0.1793 | -0.9215 | <0.0001 | 0.09386 | -0.8117 | <0.0001 |
| **PYGL** | -0.8172 | 1.78 | <0.0001 | 0.3953 | -1.85 | <0.0001 | 0.3386 | -1.08 | <0.0001 |
| **CSAD^b^** | 0.053 | -0.235 | 0.01 | 0.2330 | -1.004 | <0.0001 | ns | ns | 0.2 |
| **AKR1B1^b^** | -0.337 | 0.7925 | <0.0001 | ns | ns | 0.5 | 0.0384 | -1.295 | <0.0001 |

1. ns indicates not significant.
2. Genes involved in osmolyte synthesis
