# Supplementary material for: FLCN and AMPK Confer Resistance to Hyperosmotic Stress via Remodeling of Glycogen Stores
Source: PLoS Genet. 2015 Oct 6;11(10):e1005520. doi: 10.1371/journal.pgen.1005520 (PMC4595296; doi:10.1371/journal.pgen.1005520)
Supplement: S6 Table — (DOCX) [file pgen.1005520.s012.docx]

| **Table S6: Strains list** | | |
| --- | --- | --- |
| Genotype | Strain Number | Additional information |
| wild-type Bristol (N2) |  |  |
| *flcn-1(ok975) II* | AP2 | RB1035 strain was outcrossed 8 times with wild-type Bristol (N2) |
| *aak-1(tm1944) III* | FX1944 | from CGC |
| *aak-2(ok524) X* | RB754 | from CGC |
| *aak-2(gt33)X* | TG38 | from CGC |
| *atg-18(gk378) V* | VC893 | from CGC |
| *pmk-1(km25) IV* | KU25 | from CGC |
| *flcn-1(ok975); aak-1(tm1944)* |  |  |
| *flcn-1(ok975); aak2 (gt33)* |  |  |
| *flcn-1(ok975); atg-18(gk378)* |  |  |
| *aak-1(tm1944); aak-2(ok524)* |  |  |
| *flcn-1(ok975); aak-1(tm1944); aak-2(ok524)* |  |  |
| *flcn-1(ok975); pmk-1(km25)* |  |  |
| *gpdh-1(kb24); gpdh-2(kb33)* |  |  |
| *flcn-1(ok975); gpdh-1(kb24) I ; gpdh-2(kb33) III* |  |  |
| *flcn-1(ok975); flcn::GFP* |  | Overexpression of FLCN-1 co-injected with pRF4 (*rol-6*) and pCFJ90 (*Pmyo-2::mCherry::unc-54utr*) |
